# DCP-TS: A Unified Spatiotemporal Framework for Real-Time Desmoking and Flicker Suppression in Laparoscopic Surgical Videos

**DOI:** 10.3390/bioengineering13070714

**Published:** 2026-06-23

**Authors:** Chun-Hsien Wu, Chih-Yi Lin, Yi-Chun Du

**Affiliations:** 1 Department of Surgery, National Cheng Kung University Hospital (NCKUH), Tainan 70105, Taiwan; wuch99@gmail.com; 2 Department of Biomedical Engineering, National Cheng Kung University (NCKU), Tainan 70105, Taiwan; f94111148@gs.ncku.edu.tw; 3 Medical Device Innovation Center, National Cheng Kung University (NCKU), Tainan 70105, Taiwan

**Keywords:** surgical smoke removal, conditional generative adversarial network, dark channel prior, optical flow, temporal consistency, minimally invasive surgery

## Abstract

Surgical smoke generated by energy-based instruments during minimally invasive surgery severely degrades intraoperative visibility in laparoscopic procedures, prolonging operation time and elevating surgical risk. Although deep-learning desmoking methods have improved spatial clarity, most operate frame-by-frame and produce temporal artifacts—flicker, brightness drift, and color instability—that hinder clinical adoption. To our knowledge, no prior framework has jointly addressed spatial restoration and temporal consistency within a unified surgical smoke removal pipeline. We proposed DCP-TS, a unified spatiotemporal framework that coupled a Dark Channel Prior (DCP)-guided conditional generative adversarial network (cGAN) with an inference-time module integrating optical flow alignment, exponential moving-average luminance smoothing, and adaptive gamma correction. A key novelty was that this stabilizer was smoke-aware and operated entirely at inference time, requiring no retraining or post-processing, which distinguished it from generic video temporal-consistency methods. On laparoscopic colorectal surgery videos, DCP-TS achieved a PSNR of 23.39 dB, SSIM of 0.62, NIQE of 4.17, and BRISQUE of 23.66, outperforming DehazeFormer and Colores et al. across all metrics. Temporal analysis showed an approximate 28% reduction in inter-frame luminance variation, and a double-blind reader study with five experienced laparoscopic surgeons confirmed substantial improvements in brightness stability (4.37 vs. 2.86) and overall perceptual quality (4.18 vs. 3.51 on a 5-point Likert scale). The system ran at 22 fps with ~3.9 GB GPU memory on standard operating-room hardware, supporting real-time intraoperative deployment. DCP-TS demonstrated that physics-guided spatiotemporal modeling could transform frame-by-frame desmoking into a clinically promising, perceptually more continuous video stream.

## 1. Introduction

Colorectal cancer was the third most common malignancy and the second leading cause of cancer-related death worldwide, with approximately 1.9 million new cases and 935,000 deaths reported in 2020 [1]. By 2040, the number of cases was projected to exceed 3.2 million globally, accompanied by 1.6 million deaths [2]. In Taiwan, colorectal cancer consistently ranked first in incidence, with more than 17,000 new cases and 6000 deaths annually. It predominantly affected individuals over the age of 60 but showed an increasing trend among younger populations [3].

With the increasing disease burden, advances in surgical techniques—particularly minimally invasive approaches—became essential for improving treatment outcomes. Laparoscopic low anterior resection (LAR) and anterior resection (AR) were standard minimally invasive surgical procedures for treating colorectal cancer. Compared to open surgery, these approaches offered advantages such as reduced trauma, quicker recovery, and better preservation of anal function. However, surgical visibility was often compromised during laparoscopic operations due to smoke generated by energy-based surgical instruments in the confined pelvic cavity. Accumulated smoke and fog not only obscured the surgical field and increased the frequency of lens cleaning but also prolonged operation time and reduced surgical efficiency. Although commercial devices such as the AirSeal system or conventional smoke evacuation units were commonly used, their efficacy in managing smoke during LAR and AR remained limited.

Recent studies highlighted the importance of improving visual clarity during minimally invasive surgery. Nabeel et al. analyzed various laparoscopic lens cleaning methods, including heated scopes, anti-fog coatings, and surfactant rinsing, and reported that these techniques only provided temporary improvements and failed to address recurrent smoke accumulation [4]. Furthermore, Nabeel et al. conducted structured interviews with surgeons, which revealed that operative vision compromise (OViC) due to smoke not only increased surgical stress but also prolonged operation time and jeopardized patient safety [5]. Complementary to these findings, Łącki et al. performed a quantitative analysis of visual obstruction in minimally invasive procedures, which showed that 19–52% of operative time was spent under suboptimal visibility, with smoke accounting for approximately 53% of all obstruction events [6].

Deep learning-based desmoking methods have shown promising results in image enhancement; however, most existing models were trained on single-frame images and neglected temporal correlations between consecutive frames. Consequently, while effective in removing smoke in static images, these approaches often produced inconsistent brightness, detail loss, and noticeable flickering artifacts when applied to video sequences. Such temporal instability degraded visual quality and interfered with intraoperative decision-making, which limited the clinical applicability of these methods.

Early approaches to image dehazing and smoke removal largely relied on physical priors or statistical enhancement techniques. The Dark Channel Prior (DCP) method estimated transmission maps by observing the minimal color channels and was widely used in haze removal tasks [7]. Such prior-based methods yielded interpretable models, but in surgical or endoscopic images with specular highlights and nonuniform illumination, DCP could result in over-bright regions or color distortion. Further enhancements—such as Retinex-based illumination correction, histogram equalization, and wavelet enhancement—were applied to boost contrast, but often degraded under strong lighting variation [8,9]. Comparative studies on real-world dehazing confirmed that classical techniques lacked robustness in dense or nonhomogeneous haze conditions [10]. In medical imaging, these traditional techniques often struggled with complex light-tissue interactions, which motivated the shift to learning-based methods.

With the rise of deep learning, convolutional neural networks (CNNs) and generative adversarial networks (GANs) were adopted for smoke removal tasks. Surveys of deep learning-based dehazing presented a taxonomy of supervised and unsupervised models [11,12]. For example, deep dehazing networks using adversarial losses or residual designs showed a stronger ability to learn global and local mappings [13]. In laparoscopic image enhancement, DCP-guided conditional GANs were proposed to fuse physical priors with learning pipelines to better preserve structural consistency [14]. More recently, transformer-based architectures markedly advanced image restoration and dehazing—for example, Restormer [15] for high-resolution restoration and DehazeFormer [16] for single-image dehazing—and began to be adapted to surgical scenes, such as the local–global U-shaped transformer for endoscopic desmoking [17]. Nonetheless, most of these methods were applied frame-by-frame, without modeling temporal relationships across sequential frames. When deployed on video sequences, these per-frame approaches often produced inconsistent brightness or visible temporal artifacts.

Because each frame was enhanced independently, the network re-estimated its transmission and gain from frame-specific cues (smoke density, specular highlights, and sensor noise). In the absence of any cross-frame constraint, these per-frame estimates were temporally uncorrelated, so small input variations were amplified into brightness drift, flicker, and color instability when the outputs were viewed as a continuous video [18,19].

To mitigate these issues, video enhancement research had introduced temporal consistency regularization techniques. For instance, Bonneel et al. developed a gradient-domain method to stabilize flickering across frames [18]. Later, Lai et al. proposed a recurrent deep network to enforce temporal coherence [19]. While these techniques succeeded in general video enhancement, there remained few works that integrated deep desmoking pipelines in surgical laparoscopic scenes with these temporal stabilization modules. To the best of our knowledge, no prior work has integrated spatial smoke removal and temporal stabilization within a unified pipeline for laparoscopic surgical videos, leaving this combined framework largely unexplored.

To address these challenges, this study proposed DCP-TS, a unified spatiotemporal framework that combined a U-Net-based conditional generative adversarial network (cGAN) guided by an embedded DCP with an inference-time flicker suppression module, enabling real-time joint enhancement of spatial clarity and temporal consistency. The proposed framework integrated adaptive brightness compensation, optical-flow-guided temporal smoothing, and adaptive gamma correction directly into the inference pipeline, eliminating the need for any post-processing stage. To comprehensively evaluate its effectiveness, multiple objective image quality assessment metrics—including PSNR, SSIM, NIQE, and BRISQUE—were employed, while the AC/DC flicker ratio was used to quantitatively analyze temporal stability. Through these evaluations, DCP-TS demonstrated consistent improvements in both visual quality and brightness consistency, supporting real-time laparoscopic surgical applications.

This study offered several contributions. To the best of our knowledge, it was the first framework to couple physics-guided spatial desmoking with explicit temporal stabilization within a single real-time pipeline for laparoscopic surgical video, rather than treating the two problems separately. The proposed stabilizer was lightweight and operated entirely at inference time, combining optical-flow alignment, exponential moving-average luminance smoothing, and adaptive gamma correction, so that it required no additional training, no post-processing stage, and only about 3.9 GB of GPU memory. Importantly, the stabilization was smoke-aware, since its smoothing and fusion strengths were modulated by an estimated per-frame smoke level, which distinguished the method from generic, content-agnostic video temporal-consistency techniques. The system ran at 22 fps on laptop-class operating-room hardware, demonstrating practical intraoperative feasibility, and it was evaluated with a clinically oriented protocol that combined objective image-quality and temporal-stability metrics with a double-blind reader study by experienced laparoscopic surgeons, yielding an approximately 28% reduction in inter-frame luminance variation together with consistent perceptual gains over the desmoking-only baseline.

## 2. Materials and Methods

### 2.1. Experimental Workflow

**Figure 1:**
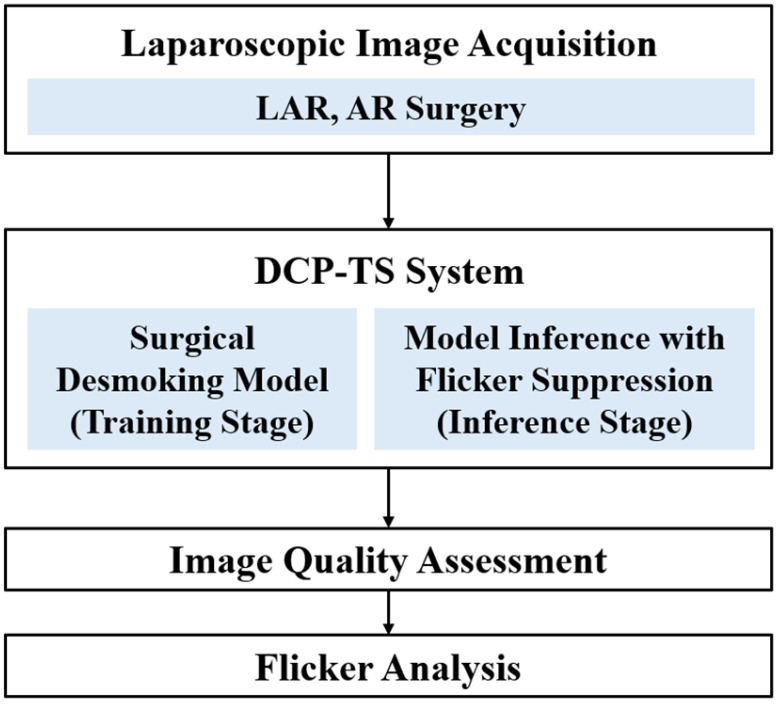
Experimental workflow of the proposed DCP-TS system for laparoscopic desmoking and flicker suppression.

The experimental workflow of the proposed system is illustrated in Figure 1. Laparoscopic video sequences obtained from LAR and AR surgeries were first collected as input data for model training and evaluation. The proposed DCP-TS framework integrated both surgical desmoking and temporal stabilization within a unified inference process. The model was built upon a DCP-guided conditional GAN that restored smoke-free tissue textures while preserving structural details. During inference, temporal stability was ensured through an integrated flicker suppression mechanism that combined optical-flow-guided feature alignment, exponential moving-average (EMA) luminance smoothing, and adaptive gamma correction. Finally, the outputs were evaluated using quantitative image quality assessment metrics for spatial and perceptual quality, as well as temporal brightness fluctuation (AC)/average brightness level (DC) ratio analysis to assess temporal consistency across frames.

### 2.2. The Framework of Surgical Desmoking Model

The proposed surgical desmoking model employed an image-to-image cGAN [20,21,22] guided by a DCP mask. The dark channel map served as an additional input channel that provided spatial cues on smoke concentration and enhanced the network’s ability to discriminate smoke-affected regions. For each laparoscopic frame It, the dark channel Idark was computed by taking the minimum intensity within a local window across RGB channels and was refined using a guided filter to preserve edge structures. The refined DCP map was then concatenated with the RGB image to form a four-channel input [R, G, B, Idark]. This embedded map directed the generator toward spatial regions requiring restoration while avoiding over-enhancement in clear areas.

The generator G adopted a U-Net [23] encoder–decoder architecture with skip connections, whereas the discriminator D was a seven-layer PatchGAN [20] classifier that evaluated local patch-level realism. The network was trained using paired synthetic datasets generated via Perlin-noise-based smoke simulation and the atmospheric scattering model. The objective function, which combined an adversarial term with an L1 reconstruction loss applied only to RGB channels, was formulated as:

G* = arg min_G_ max_D_ L_cGAN_(G, D) + λ L_L1_(G) (1)

where λ controlled the balance between adversarial realism and pixel-wise fidelity.

**Figure 2:**
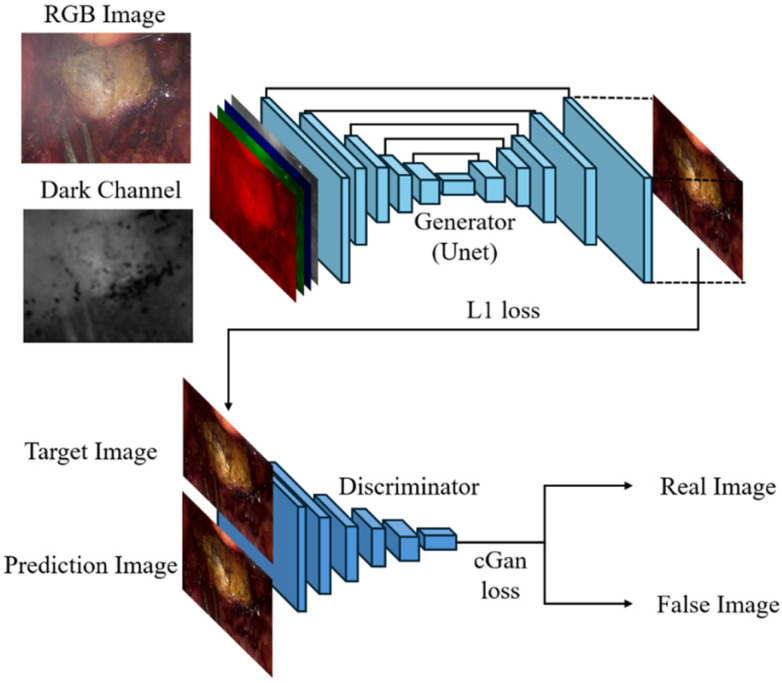
Architecture of the proposed DCP-guided conditional GAN-based desmoking model, which employs a U-Net generator and a PatchGAN discriminator to restore clear laparoscopic images.

This DCP-guided cGAN effectively recovered smoke-free tissue textures and preserved natural color tones without introducing artifacts. The trained model, as illustrated in Figure 2, served as the first stage of our DCP-TS system, providing the clean yet temporally unstabilized frames required for subsequent flicker suppression processing.

### 2.3. Flicker Suppression

**Figure 3:**
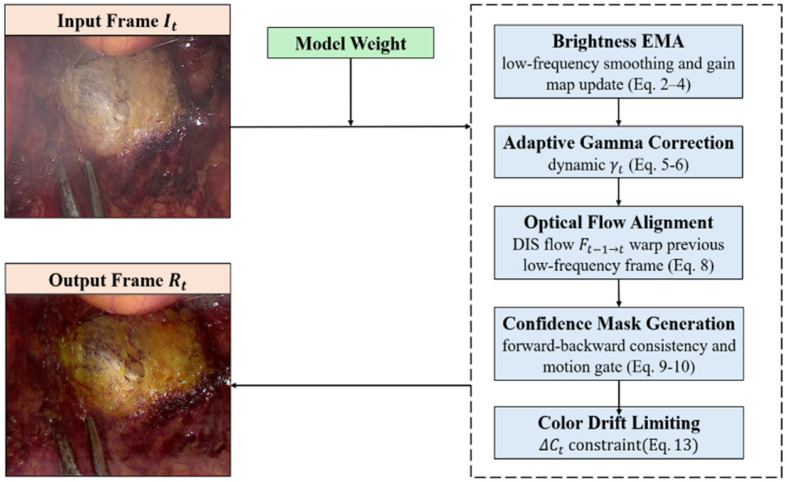
Inference-time flicker suppression pipeline.

To ensure temporal stability during inference, a flicker suppression process was integrated into the desmoking pipeline, as illustrated in Figure 3. This module compensated for frame-to-frame brightness oscillations and color drift through a combination of adaptive brightness smoothing, contrast correction, optical-flow-guided low-frequency fusion, and color drift limitation.

#### 2.3.1. Smoke-Aware Brightness Smoothing

For each desmoked frame I_t_, its Y-channel was extracted and bilateral-filtered to obtain the low-frequency brightness component L_t_. Since frame-by-frame inference could produce discontinuous luminance, an EMA was employed for temporal smoothing:

*Lˉ_t_* = *β*(*s_t_*) *Lˉ_t_*_−1_ + (1 − *β*(*s_t_*)) *L_t_* (2)

where Lˉ_t_ denoted the smoothed brightness, and β(s_t_) ∈ [0.8, 0.98] was an adaptive smoothing factor determined by the estimated smoke intensity s_t_.

The smoke level was derived from dark channel, saturation, and brightness contrast measurements. The smoke intensity s was defined as a weighted combination of the dark channel, saturation, and brightness contrast:

*s_t_* = *w*_1_ *D_t_* + *w*_2_ (1 − *S_t_*) + *w*_3_ (1 − *L_t_*) (3)

where D_t_, S_t_, and L_t_ respectively represented the dark channel, saturation, and brightness terms. A gain map was then computed as:

*G_t_* = clip(*Lˉ_t_*/*L_t_*, *g*_min_, *g*_max_) (4)

and was applied to the Y-channel to suppress exposure fluctuation while maintaining structural integrity.

#### 2.3.2. Adaptive Gamma Correction

To recover the contrast loss caused by smoke removal and EMA filtering, a dynamic gamma correction was applied:

*γ_t_* = 1 + *β_γ_* (1 − *Lˉ_t_*) (5)

where *Lˉ_t_* denoted the mean brightness of the current frame and *β_γ_* controlled enhancement intensity. The corrected image was obtained by pixel-wise tone mapping:

*I_γ_*(*i*) = 255·(*I_t_*(*i*)/255)^1/γ*t*^ (6)

#### 2.3.3. Optical-Flow-Guided Temporal Fusion

To further suppress temporal flicker, only the low-frequency components were blended across consecutive frames using dense optical flow alignment. Each corrected frame was decomposed as:

*I_t_*^L^ = *G_σ_* × *I_t_*,   *I_t_*^H^ = *I_t_* − *I_t_*^L^ (7)

where Gσ was a Gaussian low-pass kernel.

Forward and backward optical flows F_t−1_→_t_ and F_t_→_t−1_ were computed using the Dense Inverse Search (DIS) method, providing sub-pixel motion fields suitable for real-time inference. The low-frequency layer of the previous frame was warped to the current coordinate system:

*I*_*t*−1_^*L→t*^ = *W*(*I*_*t*−1_^L^, *F*_*t*−1*→t*_) (8)

and the reliability of each pixel flow was evaluated by forward–backward consistency:

*E* = ‖*F_t−_*_1_*_→t_* + *W*(*F_t__→t__−_*_1_)‖^2^ (9)

A confidence mask was then generated as:

*M_t_* = exp(−*E*/*σ*^2^) (10)

representing the occlusion-aware reliability of flow estimation.

The low-frequency fusion between current and warped previous frames was defined as:

*Ĩ_t_*^L^ = (1 − *α_t_ M_t_*) *I_t_*^L^ + *α_t_ M_t_ I_t−_*_1_*^L^^→t^* (11)

where α_t_ ∈ [0.55, 0.9] was a smoke-dependent temporal blending weight. The final temporally stabilized frame was reconstructed as:

*R_t_* = *Ĩ_t_*^L^ + *I_t_*^H^ (12)

#### 2.3.4. Color Drift Limiting

To avoid cumulative hue deviation caused by iterative fusion, color drift between consecutive frames was constrained as:

*ΔC_t_* = mean(*R_t_*) − mean(*R_t−_*_1_) (13)

and was limited by a threshold *Δ*_max_. If *ΔC_t_* > *Δ*_max_, the deviation was proportionally scaled to maintain temporal chromatic consistency. This correction prevented long-term accumulation of color bias and ensured visual naturalness.

#### 2.3.5. Theoretical Rationale and Inference Algorithm

The fusion between the DCP and the cGAN could be interpreted as spatially conditioned restoration. The dark-channel map I_dark provided a per-pixel estimate of smoke concentration; by concatenating it with the RGB image to form the four-channel input [R, G, B, I_dark], the generator received an explicit spatial prior that scaled the restoration strength with local smoke density, directing capacity toward smoke-affected regions while leaving clear areas nearly unchanged. The adversarial term in Equation (1) enforced realism, whereas the L1 term anchored pixel fidelity to the reference.

The temporal module behaved as a low-pass filter along the time axis. The EMA in Equation (2) was a first-order recursive filter whose effective time constant grew with beta(s_t); a larger beta (used under denser smoke) attenuated high-frequency luminance oscillations more strongly. The optical-flow low-frequency fusion in Equation (11) blended only the motion-compensated low-frequency band, so that static flicker was suppressed while genuine motion and high-frequency detail (I^H) were preserved; the confidence mask M_t (Equation (10)) down-weighted unreliable flow in occluded regions, and the color-drift limit (Equation (13)) prevented long-term hue accumulation. The complete inference-time procedure for a single frame was summarized in Algorithm 1.
**Algorithm 1.** Inference-time DCP-TS pipeline for one frame I_tInput: current frame I_t, previous states (Lˉ_{t − 1}, R_{t − 1}); Output: stabilized frame R_t1:  I_dark ← GuidedFilter(DarkChannel(I_t));   X ← concat([R,G,B], I_dark)2:  Î_t ← G(X)                                               # DCP-guided cGAN desmoking3:  s_t ← w1·D_t + w2·(1 − S_t) + w3·(1 − L_t)          # smoke level (Equation (3))4:  Lˉ_t ← β(s_t)·Lˉ_{t − 1} + (1 − β(s_t))·L_t            # EMA smoothing (Equation (2))5:  apply gain map G_t (Equation (4)) and adaptive gamma γ_t (Equations (5) and (6))6:  split I_t^L, I_t^H (Equation (7));   F ← DIS_OpticalFlow;   warp + mask M_t (Equations (8)–(10))7:  Ĩ_t^L ← (1 − α_t M_t) I_t^L + α_t M_t Î_{t − 1}^{L → t}       # fusion (Equation (11))8:  R_t ← Ĩ_t^L + I_t^H (Equation (12));   limit color drift ΔC_t ≤ Δmax (Equation (13))9:  return R_t

### 2.4. Evaluation Metrics

To comprehensively evaluate the proposed framework, multiple quantitative metrics were employed. Spatial image quality was assessed using Peak Signal-to-Noise Ratio (PSNR), Structural Similarity Index (SSIM) [24], Natural Image Quality Evaluator (NIQE) [25], and Blind/Referenceless Image Spatial Quality Evaluator (BRISQUE) [26].

Furthermore, temporal consistency was quantitatively analyzed using the AC/DC ratio, a brightness-stability metric designed to evaluate inter-frame luminance variation. The DC (average brightness) and AC_rms_ (brightness fluctuation) components were defined as:

DC = (1/*N*) Σ_i=1_^N^ *Y_i_*,   AC_rms_ = √((1/*N*) Σ_i=1_^N^ (*Y_i_* − DC)^2^) (14)

where *Y_i_* denoted the mean brightness of frame *i*, and *N* was the total number of frames. A lower AC/DC ratio corresponded to smoother brightness transitions and reduced flicker artifacts. The flicker ratio was then defined as:

Flicker*_t_* = AC_rms_/DC (15)

### 2.5. Dataset and Implementation Details

The dataset comprised 11 laparoscopic colorectal procedures (LAR/AR) acquired at a single center under IRB A-ER-115-049. Representative frames were sampled to reduce redundancy, yielding 6000 paired frames, which were partitioned at the patient level into training, validation, and testing subsets (approximately 70%/15%/15% of the frames, grouped by patient) to prevent leakage across splits. Paired supervision was generated by compositing Perlin-noise smoke with the atmospheric scattering model [7,27] onto clear frames, and was augmented with real smoke clips to reduce the synthetic-to-real domain gap.

Following the standard pix2pix configuration [20], the DCP-guided cGAN used a U-Net generator [23] and a seven-layer PatchGAN discriminator [20], trained with the Adam optimizer (learning rate 2 × 10^−^^4^, β1 = 0.5, β2 = 0.999) and a batch size of 1 for 200 epochs (the learning rate was held constant for the first 100 epochs and then linearly decayed to zero over the remaining 100), with the L1 weight λ = 100 in Equation (1). The guided filter [28] refined I_dark, and dense optical flow used the DIS method [29]. The temporal-module parameters were selected empirically on the validation set; their definitions and settings were summarized in Table 1.

## 3. Results

This section presented a comprehensive evaluation of the proposed DCP-TS framework, focusing on its spatial performance, temporal stability, computational efficiency, and clinical applicability. The evaluation was divided into four key aspects: (1) spatial image quality assessment using PSNR, SSIM, NIQE, and BRISQUE metrics; (2) temporal flicker suppression analysis based on the AC/DC ratio and inter-frame luminance variation; (3) runtime and memory measurements to verify real-time feasibility; and (4) a reader study involving surgical experts to assess perceptual quality and clinical usability. Together, these experiments provided a thorough validation of the framework’s quantitative superiority and practical effectiveness in enhancing laparoscopic video visualization.

### 3.1. Image Quality Assessment (IQA)

To comprehensively evaluate the spatial enhancement capability of the proposed framework, four quantitative image quality metrics were employed. Peak Signal-to-Noise Ratio (PSNR) and Structural Similarity Index (SSIM) were used to measure pixel-level fidelity and structural consistency against reference images. Additionally, the Natural Image Quality Evaluator (NIQE) and Blind/Referenceless Image Spatial Quality Evaluator (BRISQUE) were adopted to assess perceptual quality in a no-reference manner, which was particularly crucial for real-world laparoscopic scenes that lack ground-truth smoke-free images.

**Figure 4:**
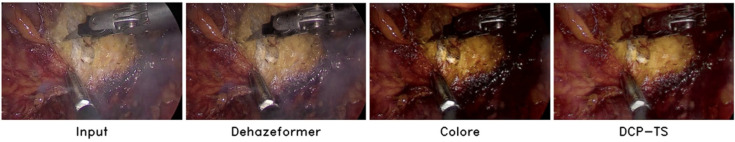
Qualitative comparison of laparoscopic desmoking results among DehazeFormer, Colores et al., and the proposed DCP-TS method. The leftmost column shows the raw, unprocessed laparoscopic input frame for reference (and the clean reference where available).

The quantitative results, summarized in Table 2, demonstrated that the proposed DCP-TS model consistently outperformed state-of-the-art methods. It achieved the highest PSNR (23.39 dB) and SSIM (0.62), indicating the best structural preservation among the compared methods. We noted that the absolute SSIM was moderate because reference-based metrics were inherently limited for real surgical scenes lacking clean ground truth; no-reference metrics (NIQE, BRISQUE) and the expert reader study (Section 3.4) therefore served as complementary evidence. Regarding no-reference perceptual quality, NIQE remained highly competitive at 4.17, while the BRISQUE score significantly decreased to 23.66, representing a substantial improvement over both Colores et al. [14] and DehazeFormer [16]. Qualitative assessments naturally aligned with these objective findings. As illustrated in Figure 4, the DCP-TS method effectively removed surgical smoke while strictly preserving natural color tones and underlying tissue textures. Compared to existing methods, the DCP-TS outputs exhibited cleaner surgical fields with significantly reduced residual haze—especially around instrument tips and tissue boundaries. This enhanced clarity successfully revealed fine vascular networks and adipose structures that remained partially obscured by other approaches. Together, these results confirmed that DCP-TS not only excelled in objective quantitative metrics but also delivered the high perceptual fidelity required for reliable clinical visualization.

### 3.2. Flicker Suppression Analysis

**Figure 5:**
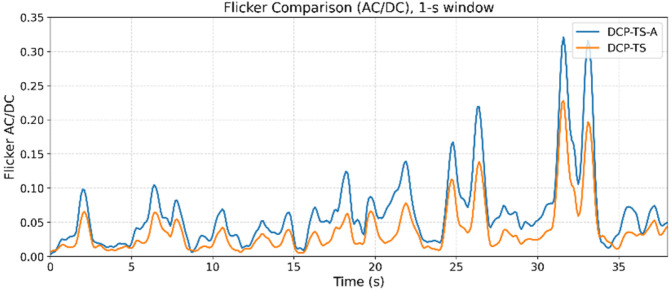
Comparison of AC/DC flicker ratio between DCP-TS-A (without temporal stabilization) and the proposed DCP-TS (with temporal stabilization). The horizontal axis denotes time in seconds (the AC/DC ratio is computed over a 1-s sliding window), and the fonts have been enlarged for clarity.

Temporal consistency was quantitatively analyzed using the AC/DC ratio to evaluate inter-frame brightness stability. As illustrated in the temporal profiles in Figure 5, the baseline DCP-TS-A model (desmoking only) exhibited noticeable fluctuations across time, reflecting severe and unstable inter-frame brightness shifts.

By contrast, the full DCP-TS system—enhanced with optical-flow alignment, exponential moving-average smoothing, and adaptive γ-correction—achieved a significantly lower and more stable AC/DC curve throughout the entire sequence, corresponding to an approximately 28% reduction in inter-frame luminance variation compared with the desmoking-only baseline. These results clearly demonstrated that the proposed temporal stabilization module effectively suppressed brightness oscillations, yielding a smoother and more coherent video output suitable for real-time laparoscopic visualization. The temporal profile spans approximately 38 s (corresponding to several hundred frames, well beyond the original short window), over which DCP-TS sustained a lower and more stable AC/DC ratio than the baseline; the baseline peaks exceeded 0.3, indicating that this value was not an upper bound.

### 3.3. Runtime and Memory

To evaluate the computational efficiency of the proposed DCP-TS system, runtime measurements were conducted on a workstation equipped with an Intel Core i7-13700H CPU, 16 GB of RAM, and an NVIDIA RTX 4050 Laptop GPU (6 GB VRAM) running PyTorch 2.5 and OpenCV 4.12. All experiments were performed on laparoscopic video sequences at a spatial resolution of 1024 × 512 pixels. The execution time of each processing stage was recorded over 3000 frames, and the results were reported as the mean ± standard deviation (SD). The total latency accounted for both the desmoking model inference and all integrated temporal stabilization modules, excluding disk I/O operations. Table 3 summarized the per-module runtime breakdown. The proposed framework achieved an average per-frame latency of 45.2 ± 5.1 ms, corresponding to an effective processing throughput of approximately 22 frames per second (fps). Within the pipeline, the desmoking generator and optical-flow alignment dominated the computational load, accounting for roughly 70% of the total runtime. Furthermore, peak GPU memory usage was constrained to approximately 3.9 GB, while CPU memory consumption remained below 1.3 GB. These performance metrics confirmed that the DCP-TS system operated well within the resource limits of standard operating room hardware, ensuring its feasibility for real-time intraoperative deployment.

Here, real-time denoted a per-frame latency low enough to provide visually continuous feedback during typical surgical maneuvers without perceptible disruption. Standard laparoscopes captured at 30–60 fps; our 22 fps (45.2 ms/frame) was therefore close to, but below, the native rate, and this gap was discussed as a limitation in Section 4.

### 3.4. Clinical Evaluation (Reader Study)

To further validate the clinical applicability and perceptual benefits of the proposed framework, a reader study was conducted involving five laparoscopic surgeons, each with over three years of relevant operative experience. Twelve anonymized video sequences (20–30 s each) were randomly selected from the test set and presented in a double-blind, randomized manner. To explicitly assess the contribution of the temporal stabilization module, each trial compared paired outputs from the ablation baseline, DCP-TS-A (desmoking only), and the full proposed system, DCP-TS (desmoking with inference-time flicker suppression). Participants evaluated four distinct visual criteria—tissue visibility, smoke cleanliness, brightness stability, and color naturalness—using a 5-point Likert scale (1 = poor, 5 = excellent).

The subjective evaluation results, summarized in Table 4, indicated that the full DCP-TS system delivered clearly better visual stability and clarity. Most notably, the integration of the temporal module yielded a substantial improvement in brightness stability, which rose from an average score of 2.86 (DCP-TS-A) to 4.37 (DCP-TS). Furthermore, tissue visibility and color naturalness saw consistent enhancements (increasing to 4.21 and 4.17, respectively), suggesting that the temporally stabilized output provided clearer operative scenes and reduced visual fatigue without introducing color distortion. Overall, the proposed framework achieved a higher average expert rating across all criteria (4.18 vs. 3.51), confirming that inference-time flicker suppression is essential for elevating frame-by-frame desmoking into a clinically reliable, perceptually continuous video stream.

### 3.5. Component Contribution and Comparison with Recent Methods

The contribution of the temporal stabilization module was supported by the comparison between the desmoking-only baseline (DCP-TS-A) and the full DCP-TS. As reported in Section 3.2, adding the module reduced the inter-frame luminance variation (AC/DC flicker ratio) by approximately 28%, and in the reader study (Table 4) it improved brightness stability from 2.86 to 4.37 and the overall expert rating from 3.51 to 4.18. A finer component-wise decomposition that isolated the individual effects of optical-flow alignment, EMA luminance smoothing, and adaptive gamma correction, together with a full quantitative parameter-sensitivity analysis, was identified as an important direction for future work.

Beyond the quantitative comparison with DehazeFormer and Colores et al. reported in Table 2, several recent methods addressed related desmoking problems, including the unpaired DeSmoke-LAP [30], the residual Swin transformer of Wang et al. [31], the self-supervised video method SelfSVD [32], and the diffusion model of Li et al. [33]. A direct quantitative benchmark against these methods was not carried out here, because publicly released weights and a common surgical benchmark were not consistently available and a fair re-implementation on our dataset was beyond the present scope; we therefore discussed them qualitatively and identified such head-to-head benchmarking as future work. Notably, the PSNR and SSIM achieved by DCP-TS (23.39 dB and 0.62) fell within the range reported by recent laparoscopic desmoking studies on real data—for example, PSNR of roughly 22–25 dB and SSIM of roughly 0.62–0.66 for DeSmoke-LAP [30] and SelfSVD [32]—where reference-based metrics remained moderate because clean smoke-free ground truth was unavailable; methods evaluated on fully synthetic paired data instead reported substantially higher values (SSIM > 0.94) that were not directly comparable.

## 4. Discussion

In this study, we proposed DCP-TS, an integrated deep learning framework designed to simultaneously perform surgical smoke removal and temporal flicker suppression in laparoscopic videos. By embedding a Dark Channel Prior (DCP)-guided conditional GAN within an inference-time stabilization pipeline—incorporating dense optical-flow alignment, exponential moving-average (EMA) brightness smoothing, and adaptive gamma correction—the system effectively enhanced both spatial clarity and temporal coherence. Quantitative evaluations demonstrated consistent improvements across all metrics; specifically, the proposed model achieved superior pixel-level fidelity (PSNR of 23.39 dB) while maintaining excellent no-reference perceptual quality (NIQE of 4.17). Furthermore, temporal analysis confirmed an approximate 28% reduction in inter-frame luminance variation, highlighting the method’s robust capability to suppress flicker artifacts and ensure stable brightness transitions. Computationally, the framework operated at an average speed of 22 fps with a moderate GPU memory footprint (~3.9 GB), validating its feasibility for real-time intraoperative deployment. Subjective evaluations by experienced laparoscopic surgeons corroborated these technical achievements, confirming that the temporally stabilized outputs provide clearer tissue visibility, smoother illumination consistency, and more natural color rendering compared to the desmoking-only baseline.

The core distinction of this work from prior art lay in its unification. Earlier studies treated spatial desmoking [7,13,14,30] and temporal consistency [18,19] as separate problems, and recent endoscopic networks [31,32,33] focused primarily on single-frame or learning-based restoration without an explicit, lightweight stabilizer. In contrast, DCP-TS (i) embedded the DCP physical prior inside the cGAN, (ii) integrated an inference-time, plug-in temporal stabilizer (optical flow + EMA + adaptive gamma) that required no retraining or post-processing and only ~3.9 GB of GPU memory, and (iii) was tailored to the clinical constraints of laparoscopic surgery. Beyond the present scope, progressive robust feature learning [34] and mask-guided multimodal fusion [35] remain promising directions for handling specular highlights and occlusions in more complex scenes.

Deep-learning-based desmoking techniques have demonstrated significant advancements in restoring spatial detail within laparoscopic videos. However, their inherent reliance on frame-by-frame enhancement frequently introduced temporal inconsistencies, such as flickering and illumination fluctuations, which remained a primary barrier to reliable intraoperative adoption. In this work, we demonstrated that integrating a physics-guided dark channel prior within a unified spatiotemporal framework provided tangible benefits that extended beyond conventional post-processing or sequential stabilization strategies. By harmonizing spatial restoration and temporal alignment within a unified inference pipeline, DCP-TS effectively improved structural clarity while strictly maintaining perceptual continuity throughout dynamic surgical scenes.

Addressing spatial–temporal degradation jointly was particularly critical in confined operative environments, where smoke and dynamic occlusions rapidly compromised visibility. By effectively preserving essential structural cues and mitigating distracting brightness shifts, DCP-TS enhanced visual certainty during video-guided manipulations, thereby potentially lowering the cognitive load on surgeons. Consequently, these observations underscored the necessity that future performance evaluations of surgical video processing systems must incorporate both objective quantitative indicators and perception-driven assessments to accurately reflect real-world clinical demands. Several limitations provided opportunities for further advancing this work. First, the present evaluation focused exclusively on laparoscopic colorectal procedures conducted at a single center. Broader multi-center studies are required to establish the generalizability of the DCP-TS framework across diverse patient anatomies, varied imaging devices, and different operative workflows. Second, although the inclusion of both real and semi-synthetic smoke enhanced model robustness, residual domain shifts between synthetic training data and real-world surgical scenes may still persist; exploring advanced unsupervised domain adaptation techniques could effectively mitigate this discrepancy. Furthermore, while the current processing speed of 22 fps provided smooth visual feedback without introducing disorienting latency for typical surgical maneuvers, it remained slightly below the standard 30 fps video capture rate. Future computational optimizations, such as network quantization and TensorRT deployment, will be pursued to further reduce inference overhead and seamlessly support higher frame rates on resource-constrained intraoperative hardware. Finally, continued clinical validation through prospective user studies will be essential to precisely quantify the magnitude of workflow improvements and safety benefits attributable to this enhanced visualization.

Limitations. Several constraints should be acknowledged explicitly. (1) The data originated from a single center and a single procedure type; multi-center validation across devices and anatomies is needed. (2) The reported SSIM (0.62) was moderate; because real surgical frames lacked clean ground truth, reference-based metrics were inherently limited, which is why the no-reference metrics and the expert reader study were emphasized. (3) The system ran at 22 fps (45.2 ms/frame) on a laptop-class RTX 4050, below the 30–60 fps native capture rate, which may introduce latency during precise maneuvers; TensorRT deployment and network quantization are expected to reach ≥30 fps. (4) Processing was performed at 1024 × 512 obtained by proportional resizing with padding (not anatomical cropping), so that true anatomical proportions were preserved; nonetheless, native-resolution and native-aspect-ratio processing remains future work. (5) A residual synthetic-to-real domain gap may persist, motivating unsupervised domain adaptation. (6) The present evaluation did not yet include a full component-wise ablation, an exhaustive parameter-sensitivity analysis, a quantitative head-to-head benchmark against the most recent methods, or formal statistical uncertainty estimates (confidence intervals and significance tests); these are acknowledged as important directions for future work. (7) The AC/DC flicker ratio is aligned with the objective of the temporal module, so the observed reduction should be read as evidence that the module behaved as intended rather than as an independent confirmation of clinical benefit; an independent temporal-consistency measure on motion-containing segments (e.g., a warping error) is planned to corroborate it. (8) Because the module performs low-pass luminance smoothing and low-frequency motion-compensated fusion, it may attenuate genuine rapid changes such as sudden bleeding or fast instrument motion; this stability-versus-responsiveness trade-off was not yet quantified on high-motion segments. (9) Reference-based metrics (PSNR/SSIM) were necessarily computed on synthetic smoke pairs for which a clean reference exists, whereas the perceptual and clinical claims pertain to real footage, so the two were not measured on the same distribution, and the realism of the synthetic smoke model is itself a limitation. (10) The reader study compared the full system against its desmoking-only ablation rather than against the unprocessed video or competing methods, and used a small panel without inter-rater agreement or task-based outcome measures; it should therefore be interpreted as evidence for the contribution of the temporal module rather than as a clinical validation. (11) Although the temporal stabilizer is presented within a unified pipeline, it is method-agnostic; a direct comparison against an existing desmoker combined with a generic video temporal-consistency method [18,19] would better isolate the benefit of the proposed joint design and is left for future work.

In summary, unlike conventional physical lens-cleaning methods that offered only temporary relief, DCP-TS introduced a unified, real-time spatiotemporal enhancement strategy that simultaneously mitigated smoke interference and ensured brightness stability across laparoscopic video streams. The demonstrated perceptual improvements and operational feasibility strongly supported its potential role as an intraoperative visualization assistance tool, ultimately ensuring continuous, reliable, and clear surgical visibility.

## 5. Conclusions

Overall, the DCP-TS framework effectively integrated spatial enhancement and temporal stabilization in a unified pipeline, delivering both quantitative and perceptual improvements that enhanced intraoperative visualization and supported real-time clinical applications in minimally invasive surgery. By coupling a Dark Channel Prior-guided cGAN with inference-time optical-flow alignment, EMA luminance smoothing, and adaptive gamma correction, DCP-TS achieved superior pixel-level fidelity (PSNR of 23.39 dB), an approximately 28% reduction in inter-frame luminance variation, and consistently higher expert ratings (4.18 vs. 3.51 on a 5-point Likert scale) compared with the desmoking-only baseline, while operating at 22 fps with a moderate GPU memory footprint of approximately 3.9 GB. Taken together, these results indicate that physics-guided spatiotemporal modeling converts per-frame desmoking into a temporally coherent video stream, supporting broader multi-center validation and integration into next-generation intraoperative visualization systems.

## Figures and Tables

**Table 1:** Mathematical symbols and their definitions.

Symbol	Description	Typical Value/Range
I_t	input laparoscopic frame at time t	RGB image
I_dark	refined dark-channel map (guided-filtered)	single-channel map
L_t, Lˉ_t	instantaneous/EMA-smoothed luminance	[0, 1]
s_t	estimated smoke level	[0, 1]
D_t, S_t	dark-channel and saturation terms	[0, 1]
β(s_t)	adaptive EMA smoothing factor	0.80–0.98 (adaptive)
w1, w2, w3	weights in smoke-level estimate	0.5, 0.2, 0.3
G_t	luminance gain map (clipped to [g_min, g_max])	g_min = 0.8, g_max = 1.2
γ_t, βγ	adaptive gamma and its intensity	βγ = 0.6
I_t^L, I_t^H	low-/high-frequency bands (Gaussian split, σ)	σ = 1.5
F	forward/backward optical flow (DIS)	2-D vector field
M_t	forward–backward confidence mask	[0, 1]
α_t	temporal fusion weight	0.55–0.90 (adaptive)
R_t	temporally stabilized output frame	RGB image
ΔC_t, Δmax	inter-frame color drift/threshold	Δmax = 5
λ	L1 weight in the cGAN objective	100

**Table 2:** Quantitative image quality comparison. ↑ indicates that higher values are better; ↓ indicates that lower values are better.

Metrics	DehazeFormer	Colores et al.	DCP-TS
PSNR ↑	19.65	22.50	23.39
SSIM ↑	0.57	0.60	0.62
NIQE ↓	4.89	4.35	4.17
BRISQUE ↓	33.93	27.17	23.66

**Table 3:** Per-module latency and memory usage of the DCP-TS system.

Module	Mean (ms)	SD (ms)	Share (%)
Desmoking (U-Net cGAN, FP16)	19.6	2.4	43.4
Brightness EMA & Gain Map	1.8	0.4	4.0
Adaptive Gamma Correction	0.9	0.2	2.0
DIS Optical Flow Computation	11.7	2.3	26.0
Warping & Luminance Matching	3.1	0.6	6.9
Confidence Mask Generation	1.2	0.3	2.7
Low-Frequency Fusion & Blending	1.0	0.3	2.2
Color Drift Limiting	0.6	0.1	1.3
Other (color conversion, host–device transfer, and overhead)	5.3	0.5	11.5
Total	45.2	5.1	100.0

**Table 4:** Average ratings on twelve video pairs with five experts.

Criterion	DCP-TS-A	DCP-TS
Tissue visibility	3.78	4.21
Smoke cleanliness	3.68	3.98
Brightness stability	2.86	4.37
Color naturalness	3.74	4.17

## Data Availability

The laparoscopic surgical video datasets analyzed in this study are not publicly available due to patient privacy considerations and institutional restrictions associated with the IRB-approved protocol (A-ER-115-049). De-identified data subsets supporting the findings of this study are available from the corresponding author (Y.-C.D.) upon reasonable request and subject to approval by the National Cheng Kung University Hospital Institutional Review Board.
